# Relationship between Cardiometabolic index and endometriosis in a US nationally representative sample: results from NHANES 1999-2006

**DOI:** 10.3389/fendo.2024.1450965

**Published:** 2024-11-12

**Authors:** Jiarui Hou, Wenting Chen, Rui Wang, Xuchun Huang, Xiaojing Cao, Xiaoyun Wang

**Affiliations:** ^1^ The Second Clinical College of Guangzhou University of Chinese Medicine, Guangzhou, China; ^2^ Department of Gynecology, The Second Affiliated Hospital of Guangzhou University of Chinese Medicine, Guangzhou, China

**Keywords:** endometriosis, cardiometabolic index, epidemiology, cross-sectional study, NHANES (National Health and Nutrition Examination Survey)

## Abstract

**Background:**

Endometriosis is an estrogen-dependent gynecological endocrine condition and a systemic inflammatory disease associated to improper lipid metabolism and increased cardiovascular risk. The Cardiometabolic Index (CMI) is a novel indicator representing visceral adipose tissue distribution and metabolic dysfunction, integrating lipid metabolism indicators and the waist-to-height ratio. While anomalies in lipid metabolism are often associated with (BMI) Body Mass Index, literature consistently shows a negative link between endometriosis and female BMI, and some studies have found that endometriosis is one of the few reproductive diseases not persistently positively correlated with obesity. Given the limitations of BMI, a comprehensive index like CMI is crucial for exploring the incidence of endometriosis. Currently, research on the correlation between CMI and endometriosis is lacking, prompting this study to investigate this association.

**Objective:**

To investigate the association between the CMI and the risk of having endometriosis in a sample representing the entire U.S. population.

**Study design:**

A cross-sectional analysis was conducted using data from four cycles of the National Health and Nutrition Examination Survey (NHANES) spanning the years 1999 to 2006. The study included individuals aged 20 to 54 with a documented history of endometriosis and complete CMI data. Logistic regression analysis, subgroup and interaction analyses, smooth curve fitting, and restricted cubic splines (RCS) were utilized to examine the association between CMI and endometriosis.

**Results:**

The study found that individuals with higher CMI had an increased probability of developing endometriosis. This relationship remained significant after adjusting for potential confounders such as age, ethnicity, Poverty Income Ratio (PIR), drinking, smoking, education level, and marital status. The fully adjusted model revealed a positive correlation between CMI and endometriosis (OR = 1.21; 95% CI, 1.04–1.40, p < 0.05). Subgroup and interaction analyses showed no significant effect modification by age, BMI, PIR, hypertension, drinking, smoking, or menarche age (all p-values for interaction > 0.05).

**Conclusion:**

Our study shows a link between CMI and the chance of getting endometriosis.Due to the common occurrence of endometriosis and the lack of clarity surrounding their cause, more study is needed to confirm our results and find out if CMI could be used as a warning sign for endometriosis.

## Introduction

Endometriosis is a condition in which endometrial tissue (including glands and stroma) is found outside the uterus. It typically manifests as dysmenorrhea, menstrual irregularities, chronic pelvic pain, and infertility. Endometriosis exhibits biological characteristics similar to those of malignant tumors, such as invasiveness, implantation, and recurrence. Additionally, endometriosis provide a potential threat of long-term development of cancer (1). Over the past few years, the occurrence of endometriosis has been steadily rising, with a worldwide occurrence rate ranging from 10% to 15% (2). Studies indicate that the average diagnostic delay for endometriosis is 7-11 years (3). Delayed diagnosis, physical discomfort, hormonal therapy, and inconvenience in daily life and work significantly reduce the quality of life for endometriosis patients. Nevertheless, the underlying cause of endometriosis is still not fully understood. Recently, scientists have enhanced their comprehension of endometriosis, acknowledging it as both an estrogen-dependent gynecological endocrine condition and a systemic inflammatory disease associated to irregularities in lipid metabolism and a heightened susceptibility to cardiovascular disorders (3).

Contemporary studies suggest that anomalies in lipid metabolism are frequently associated with BMI (4). However, existing literature consistently demonstrates a negative link between the occurrence of endometriosis and female BMI (5–7). Additionally, some studies, such as a Mendelian randomization study, have found that endometriosis is one of the few reproductive diseases not persistently positively correlated with obesity (8). Therefore, employing a more comprehensive and scientific composite index to explore the incidence of endometriosis is crucial.

The cardiometabolic index (CMI) was initially introduced in 2015 (9). It is regarded as a novel measure that indicates the distribution of visceral fat and metabolic dysfunction.effectively integrating anthropometric and biochemical parameters, including TG, HDL-C, and WHtR, making individual parameters more holistic and comprehensive. Research has demonstrated that CMI can function as an innovative indicator of cardiovascular risk, allowing for the prediction of stroke outcomes in the general population (10). It is also associated with hypertension, diabetes, depression, and other diseases (11–13). Currently, there is a lack of research investigating the correlation between CMI and endometriosis. Hence, this study seeks to conduct an initial investigation into the correlation between CMI and endometriosis.

## Methods

### Data source and study design

We performed a cross-sectional analysis utilizing data from the NHANES database. NHANES uses a stratified, multistage probability sampling technique to track the health and nutritional well-being of adults and children in the United States. The National Center for Health Statistics (NCHS), which is under the Centers for Disease Control and Prevention (CDC), administers this program. It involves conducting physical examinations and household interviews (14). We employed data from four survey cycles spanning an eight-year duration (1999-2006). The NHANES study protocol received approval from the NCHS Research Ethics Review Board, and all participants granted signed informed consent.

**Figure 1:**
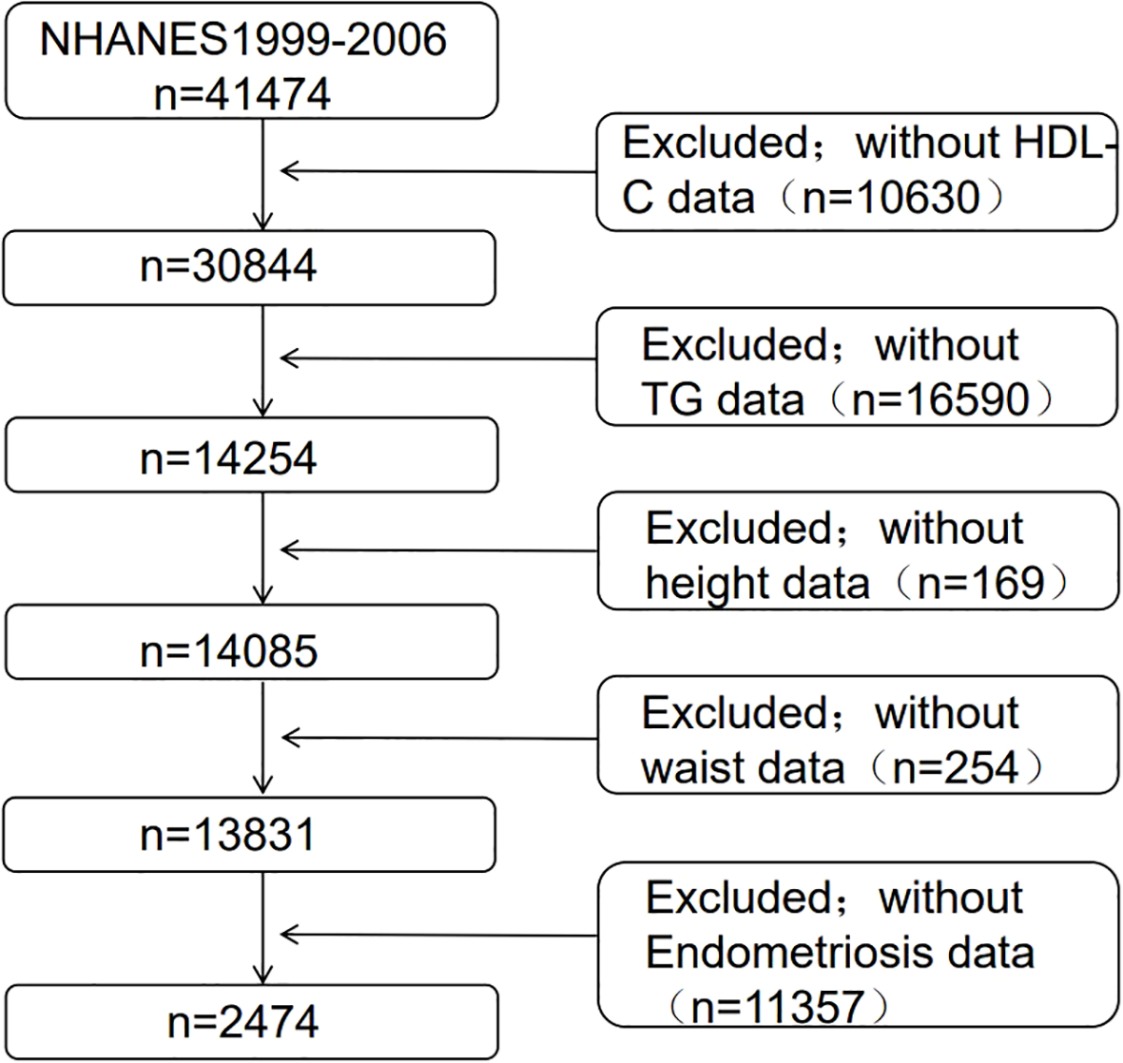
Clinical characteristics of the study population grouped by endometriosis.

This study included 41,474 participants. We selected female participants aged 20-54 years, adhering strictly to exclusion criteria: (1) lack of interview data regarding endometriosis (n=11,357), and (2) incomplete data on TG (n=16,590), HDL-C (n=10,630), waist circumference (n=254), and height (n=169). In our final analysis, a total of 2,474 eligible participants were included. Among them, 182 persons were diagnosed with endometriosis by a doctor, while 2,292 participants did not have this diagnosis (**Figure 1**).

### Cardiometabolic index

The CMI is a novel indicator derived from a combination of lipid and obesity-related parameters. It is calculated based on anthropometric data and laboratory evidence. Blood samples are usually obtained at survey trucks or specific sampling locations, where measures of TG and HDL-C are taken. The measurement of TG is restricted to specimens obtained from people who have undergone a minimum fasting period of 9 hours prior to venipuncture. This requirement guarantees the preservation and stability of the stored samples. The samples undergo laboratory processing and are submitted to thorough testing following a standardized sampling approach to guarantee the quality and comparability of the data. Trained health technicians use mobile screening equipment to measure the height and waist circumference of participants. In our study, CMI is regarded as an independent variable. The formula for calculating CMI is as follows:


(1) WHtR = waist circumference (cm)/height (cm);

(2) CMI = TG (mmol/L)/HDL−C(mmol/L) × WHtR.

### Endometriosis

During the reproductive health examination conducted at the Mobile Examination Center (MEC), individuals between the ages of 20 and 54 were privately interviewed to determine if a doctor or other healthcare provider had previously diagnosed them with endometriosis. Endometriosis is a medical disorder characterized by the presence of endometrial tissue, which is identical to the lining of the uterus, growing outside the uterus, specifically on the ovaries or fallopian tubes. Based on this information, we generated a binary variable to indicate if there was a history of endometriosis diagnosis (yes or no). The outcome variable in our study is endometriosis.

### Covariates

In our analysis, we additionally included factors that could potentially affect the relationship between CMI and endometriosis. The selection of these covariates was based on pertinent literature about the exposure and outcome variables (15–17). The variables considered in this study are age, ethnicity (specifically Mexican American, Other Hispanic, Non-Hispanic White, Non-Hispanic Black, and Other Race), education level (categorized as less than high school, high school, and college or higher), and the ratio of family income to poverty (PIR). PIR is determined by dividing the total family income by the official poverty threshold and is further classified into low (<1.3), intermediate (≥1.3 and <3.0), and high (≥3.0) groups. Other variables consist of smoking status (current smoker and non-smoker), alcohol consumption (defined as an average daily intake exceeding healthy limits, specifically more than one drink per day for women) (18), marital status (married/living with partner, separated/divorced/widowed, and never married), and whether participants had undergone a hysterectomy.

### Statistical analysis

In order to guarantee that the survey results accurately reflect the entire nation, the analysis took into consideration sampling weights and the intricate sample structure to adjust for the overrepresentation of specific population groupings. Categorical variables were represented as percentages, whereas continuous variables were presented as the mean plus or minus the standard deviation (SD). Multiple imputation techniques were employed to address the absence of covariate data (hysterectomy missing data: 1300, alcohol consumption missing data: 874, smoking missing data: 1519, marital status missing data: 72, education missing data: 2, and PIR missing data: 160). We employed logistic regression analysis models to investigate the correlation between CMI and endometriosis, and evaluated the connection between the four constituents of CMI (TG, HDL-C, waist circumference, and height) and endometriosis. In the models, CMI, TG, HDL-C, waist circumference, and height were treated as continuous variables.Model 1 did not take into account any covariates. Model 2 incorporated age, ethnicity, and PIR as covariates. Model 3 went a step further and included age, ethnicity, PIR, alcohol consumption, smoking, education level, marital status, and hysterectomy as covariates. Furthermore, the researchers did subgroup analyses to examine the relationship between CMI and endometriosis.These analyses involved categorizing the participants based on several characteristics such as age, BMI, PIR, hypertension, alcohol use, smoking, and age at first menstruation (menarche). These stratification criteria were selected as potential effect modifiers. Interaction terms were included in the model to assess the variability of the relationship between CMI and endometriosis across different subgroups.In order to examine the possible linear or nonlinear relationship between CMI and endometriosis, we utilized smooth curve fitting and limited cubic spline (RCS) analysis.The data analyses were conducted using R version 3.4.3 (http://www.R-project.org, The R Foundation) and Empower software (www.empowerstats.com; X&Y solutions, Inc., Boston, MA). The threshold for statistical significance was established at a p-value of less than 0.05.

## Results

### Baseline characteristics

An analysis of descriptive statistics was performed on a sample of 2474 participants in order to compare the sociodemographic and laboratory data features of persons with and without endometriosis(**Table 1**). No statistically significant differences were observed in BMI, HDL-C, height, smoking, and drinking habits between participants with endometriosis and those without the condition (p > 0.05). Individuals diagnosed with endometriosis exhibited several distinguishing characteristics compared to those without endometriosis. On average, they were older, experienced menarche at an earlier age, had higher levels of TG, possessed a larger waist circumference, were more likely to be Non-Hispanic White, had attained higher levels of education (high school and beyond), had a relatively higher PIR, were married or cohabiting with a partner, had higher blood pressure, and a greater proportion of them had elevated CMI. Furthermore, the percentage of oophorectomy and hysterectomy was greater in the group with the disease compared to the group without the condition (p < 0.05).Additionally, to gain a more comprehensive understanding of the sample, the characteristics of excluded and included female samples were compared (**Supplementary Table S1**).

**Table 1:** Basic characteristics of study participants from NHANES 1999–2006.

characteristics	Total	Endometriosis		P for trand
		With	Without	
Sample size	2274	182	2292	
**Age (years)**	37.17 ± 9.95	40.37 ± 8.22	36.82 ± 10.06	<0.0001
**BMI (kg/m^2^)**	28.03 ± 7.09	28.56 ± 6.66	27.98 ± 7.14	0.2249
**Menarche age (years)**	12.65 ± 1.66	12.42 ± 1.68	12.68 ± 1.66	0.0245
**TG (mmol/L)**	1.38 ± 1.19	1.84 ± 2.48	1.33 ± 0.93	<0.0001
**HDL-C (mmol/L)**	1.47 ± 0.41	1.44 ± 0.42	1.47 ± 0.40	0.2839
**waist circumference (cm)**	91.99 ± 16.00	94.06 ± 15.36	91.76 ± 16.05	0.0338
**height (cm)**	163.46 ± 6.63	163.77 ± 6.07	163.43 ± 6.69	0.4498
**Ethnicity,%**				<0.0001
Mexican American	7.69	1.48	8.37	
Other Hispanic	5.81	1.78	6.25	
Non-Hispanic White	68.83	84.70	67.12	
Non-Hispanic Black	12.42	8.36	12.85	
Other Race	5.25	3.67	5.42	
**Education level,%**				0.0057
Less than high school	15.98	10.16	16.61	
High school	23.24	29.58	22.55	
More than high school	60.78	60.26	60.83	
**PIR,%**				0.0149
Low	21.58	20.57	21.68	
Intermediate	27.12	20.08	27.89	
High	51.30	59.35	50.43	
**marital status,%**				0.0002
Married/living with partner	66.60	75.19	65.67	
Separated/divorced/widowed	15.35	16.32	15.24	
Never married	18.06	8.49	19.09	
**Blood pressure (mmHg)**				
systolic	113.98 ± 14.34	117.34 ± 15.72	113.62 ± 14.14	0.0001
diastolic	69.82 ± 10.05	72.44 ± 10.40	69.54 ± 9.96	<0.0001
**Smoking,%**				0.5840
Current-smoker	52.84	54.50	52.65	
Non-smoker	47.16	45.50	47.35	
**Drinking,%**				0.2504
Yes	61.71	58.30	62.08	
No	38.29	41.70	37.92	
**Oophorectomy (at least one ovary), %**				<0.0001
Yes	10.00	40.28	6.68	
No	90.00	59.72	93.27	
**hysterectomy, %**				<0.0001
Yes	19.7	48.38	16.57	
No	80.3	51.62	83.43	
**CMI**	0.65 ± 0.86	0.95 ± 1.77	0.62 ± 0.69	<0.0001

Data are presented as (%) or Mean ± SD.

BMI, body mass index; TG, triglycerides; HDL-C, high-density lipoprotein cholesterol; PIR, Poverty income ratio.

### Association between CMI and endometriosis

**Figure 2:**
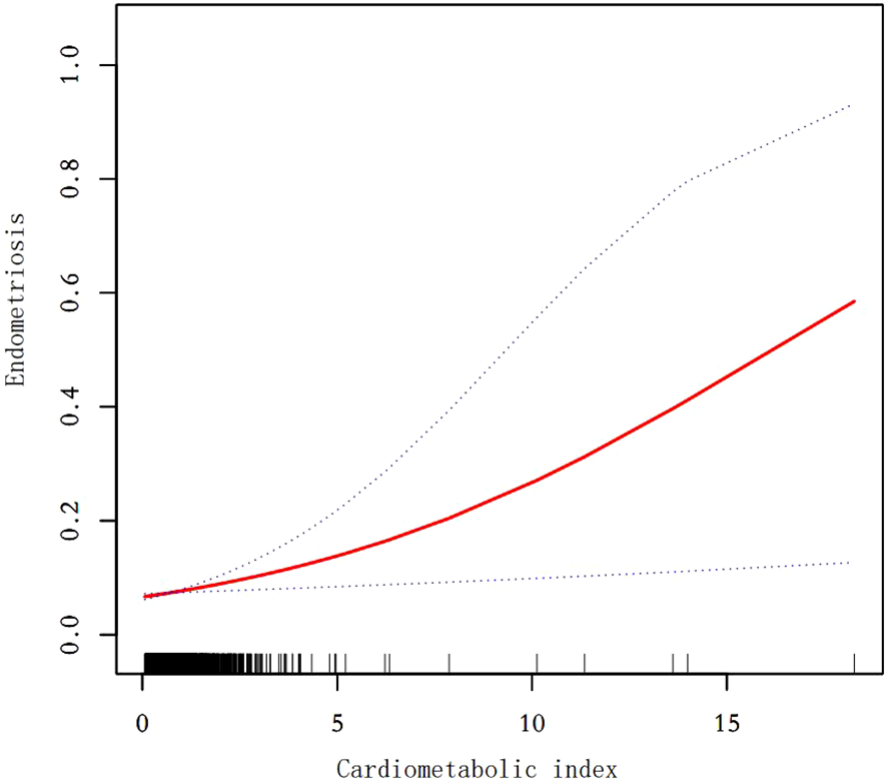
Smooth curve fit between cardiometabolic index and endometriosis. The solid red line represents the smooth curve fit between variables. Blue bands represent the 95% confidence interval from the fit. After adjusting for age,ethnicity,PIR,drinking,smoking,education level,marital status,hysterectomy, the relationship between cardiometabolic index and endometriosis was analyzed by smooth curve fitting.

**Figure 3:**
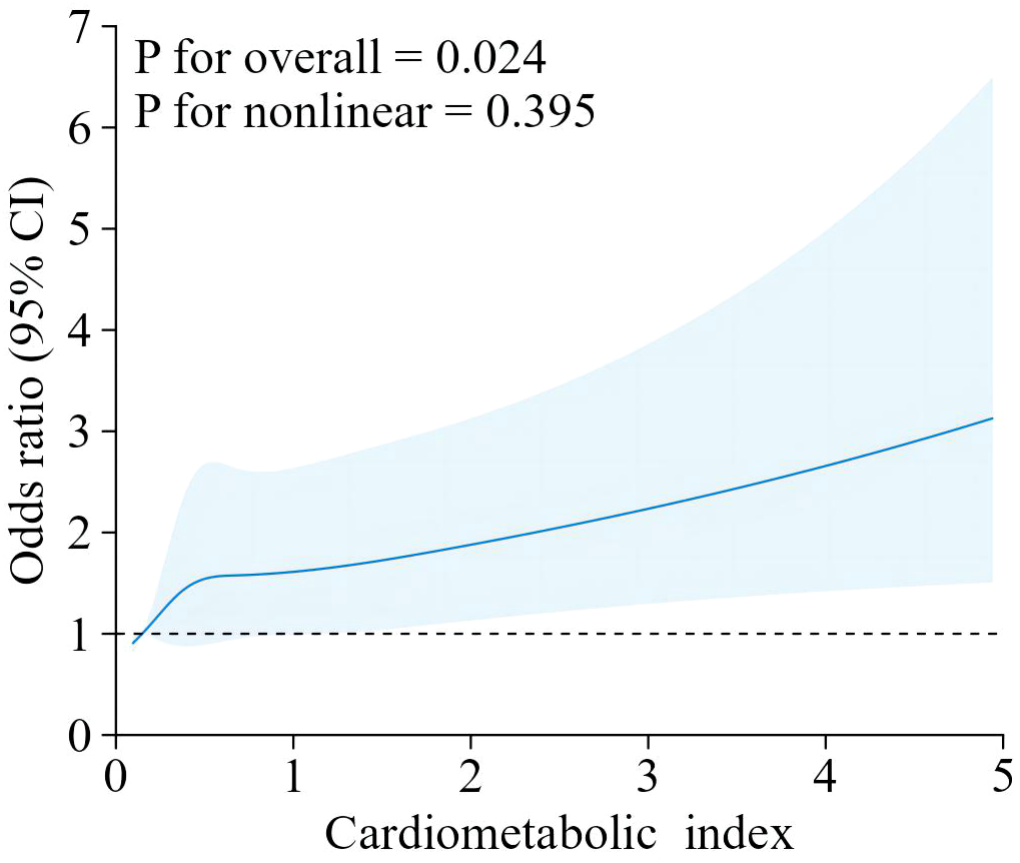
The RCS curve of the association between cardiometabolic index and endometriosis among all the study participants.RCS regression was Adjusted for age, ethnicity, PIR, drinking, smoking, education level, marital status,hysterectomy.

Our findings suggest that there is a positive correlation between CMI and endometriosis (**Table 2**). In the model that accounted for all relevant factors, the correlation between CMI and endometriosis remained consistent and positive (OR = 1.21; 95% CI, 1.04–1.40, p < 0.05). This means that for every unit increase in CMI, there was an 21% higher likelihood of experiencing endometriosis. Since CMI is derived from four parameters, we performed a logistic regression analysis to further investigate TG, HDL-C, waist circumference, and height. The findings indicated a positive correlation between TG and the occurrence of endometriosis. In the model that accounted for all relevant factors, the correlation between TG and endometriosis remained consistent and positive (OR = 1.18; 95% CI, 1.05–1.32, p < 0.05). Nevertheless, there was no statistically significant association found between HDL-C, waist circumference, and height (p > 0.05). Furthermore, the findings from the smoothed fitting curve confirmed the positive correlation between CMI and endometriosis (**Figure 2**). We also employed restricted cubic splines in the regression model to analyze the correlation between CMI and endometriosis. Despite the absence of a nonlinear relationship (nonlinearity P = 0.395), a significant positive association between CMI and endometriosis was observed (overall P < 0.05) (**Figure 3**).

**Table 2:** Associations between cardiometabolic index and endometriosis.

	OR (95% CI)		
	Model 1	Model 2	Model 3
**CMI**	1.14 (1.01,1.29)	1.17 (1.03,1,32)	1.21 (1.04,1.40)
**TG (mmol/L)**	1.13 (1.02,1.24)	1.15 (1.04,1.28)	1.18 (1.05,1.32)
**HDL-C (mmol/L)**	0.95 (0.66,1.37)	0.87 (0.60,1.27)	0.91 (0.62,1.35)
**waist circumference (cm)**	1.00 (1.00,1.01)	1.01 (1.00,1.02)	1.01 (1.00,1.02)
**height (cm)**	1.02 (1.00,1.04)	0.99 (0.96,1.01)	0.98 (0.96,1.01)

Model 1: no covariates were adjusted.

Model 2: Adjusted for Age, Ethnicity,PIR.

Model3: Adjusted for Age, Ethnicity, PIR, drinking, smoking, Education level, marital status, hysterectomy.

### Subgroup analyses

We conducted subgroup analyses to evaluate the consistency of the relationship between CMI and endometriosis in different demographic groups. To assess any potential variations in this relationship, we included an interaction term to test for heterogeneity of associations across subgroups (**Table 3**). The results revealed that there were no statistically significant interaction-related p-values, indicating that there was no evidence of effect modification by the examined factors (age, BMI, PIR, hypertension, drinking, smoking, and age of menarche) (all interaction p-values > 0.05). However, this does not imply that the relationship between CMI and endometriosis was unaffected by these factors, as confounding rather than effect modification may explain the consistent associations observed across subgroups. The correlation between CMI and endometriosis remained statistically similar across various subgroups, suggesting its relevance in diverse population settings.

**Table 3:** Subgroup analysis of the association between cardiometabolic index and endometriosis.

Subgroup	OR (95% CI)	P for interaction
**Age**		0.767
<35	1.29 (0.76,2.17)	
≥35	1.18 (1.03,1.34)	
BMI		
<25	1.79 (1.01,3.16)	0.1056
25-29.9	1.16 (0.78,1.73)	
≥30	1.03 (0.84,1.27)	
**PIR,%**		0.4944
Low	1.26 (1.02,1.56)	
Intermediate	1.01 (0.70,1.47)	
High	1.12 (0.91,1.38)	
**Hypertension**		0.9395
Yes	1.17 (0.74,1.85)	
No	1.19 (1.03,1.37)	
**Drinking**		0.9510
Yes	1.17 (1.02,1.31)	
No	1.16 (0.88,1.53)	
**Smoking**		0.7106
Yes	1.15 (1.00,1.32)	
No	1.23 (0.89,1.69)	
**Menarche age (years)**		0.4988
<13	1.12 (0.94,1.34)	
≥13	1.23 (1.01,1.49)	

## Discussion

In our cross-sectional study comprising 2474 participants, we identified a positive link between CMI and endometriosis. Specifically, individuals with elevated CMI levels had a higher likelihood of being diagnosed with endometriosis. This association remained largely unaffected by potential confounding factors such as age, Ethnicity, PIR, drinking, smoking, Education level, marital status and hysterectomy. Even after accounting for several factors that could influence the results, the fact that these connections remain independent highlights the strength and reliability of the observed link.

Moreover, prior research has shown that lipid profiles associated to atherosclerosis can be detected in both the plasma and peritoneal fluid (PF) of women with endometriosis (19), suggesting plasma component exudation during PF formation (20). This finding provides evidence for the concept that women with endometriosis may also experience a higher occurrence of dyslipidemia. In 2010, Melo et al. conducted a case-control study to assess lipid metabolism-related markers in the blood of patients with endometriosis and normal women who had not undergone hormone therapy for a minimum of three months (21). For the first time, they found elevated TG in the serum of endometriosis patients. For this investigation, we employed CMI as a composite indicator. To ensure its specificity, we conducted a logistic regression analysis on TG, HDL-C, waist circumference, and height. The results indicated a similar association between CMI and endometriosis compared to the correlation between TG and endometriosis. However, the specific mechanism underlying the connection between CMI and endometriosis remains unclear. There are three possible explanations supporting our results. First, it may be related to chronic inflammation. Previous studies have well-documented the correlation mechanisms between obesity and inflammation (22). Additionally, lipid metabolism in adipose tissue remains dysregulated during inflammation, characterized by massive infiltration of pro-inflammatory cells and high cytokine concentrations (23). The NF-κB signaling system is essential in the development of lipid metabolic problems that occur with obesity (24). Recent research indicates that in the inflammatory conditions associated to endometriosis, there are notable alterations in many cytokines at both the serum and local levels. These changes are particularly evident in IL-6, IL-8, and IL-1β, along with anomalies in the innate immune system,While numerous signaling pathways are believed to contribute to the inflammatory process associated with endometriosis, only NF-κB has been conclusively demonstrated to be implicated (25). Thus, it is postulated that the correlation between CMI and endometriosis may be associated to the NF-κB signaling pathway. Elevated CMI levels may indicate a persistent inflammatory condition within the body and can activate the NF-κB signaling pathway, potentially resulting in the proliferation of endometrial tissue outside the uterus and the development of lesions known as endometriosis. Additionally, it could be associated with atypical hormone levels. Typically, overweight individuals who are capable of having children have a significant amount of fatty tissue, which has a crucial function in the body’s steroid metabolism. Elevated aromatase activity in adipose tissue results in heightened conversion of androgens into estrogens.Therefore, obesity is closely associated with elevated estrogen levels. This elevation in estrogen levels may disrupt the balance of the endocrine system (26). At the same time, the occurrence of endometriosis involves a series of biological processes, one of which is the growth of ectopic endometrial implants (27). This process is estradiol-dependent, an estrogen steroid hormone produced not only in the ovaries but also locally, including in endometrial lesions through steroidogenic enzymes such as aromatase (28, 29). Estradiol stimulation leads to the production of prostaglandins, forming a feed-forward mechanism. Ectopic endometrial tissue overexpresses estrogen receptor β (ER-β), which suppresses estrogen receptor α (ER-α), reducing the induction of ER-α-mediated prostaglandin receptors. Ultimately, the overexpression of ER-β promotes cell survival and maintains the persistence of inflammation (30, 31). Therefore, it is speculated that elevated CMI may affect estrogen secretion, which may in turn influence the growth and migration of endometrial tissue, leading to the occurrence of endometriosis. Third, it could be associated with atypical vascular functionality. The development of adipose tissue involves a diverse range of cell types, such as adipocytes, adipose stromal cells (ASCs), endothelial cells, and inflammatory cells (32). The presence of diverse cell populations with different characteristics influences the production of various growth factors and cytokines, which have the ability to separately or together control the process of angiogenesis (33). Adipocytes in the process of growth create numerous substances that promote the formation of new blood vessels, such as leptin, VEGF, FGF-2, HGF, IGF, TNF-α, TGF-β, placental growth factor (PlGF), VEGF-C, resistin, tissue factor (TF), neuropeptide Y (NPY), heparin-binding EGF-like growth factor, and Angs (34). Pre-adipocytes and adipocytes also synthesize lipids, including monobutyrin, which promote angiogenesis in adipose tissue (35, 36). The cause of endometriotic lesions is still debated, but it is clear that the growth and survival of these lesions heavily rely on the development of new blood vessels. These blood vessels provide oxygen and necessary nutrients for the lesions to thrive (37–40). Endometriosis lesions are typically characterized by extensive angiogenesis (41). Thus, there is speculation that a high CMI may indicate the development of compact blood vessels. These irregularities can impact the blood flow and circulation in the endometrium, therefore affecting the development of endometriosis.

The present investigation possesses numerous advantages. The study initially included nationally representative NHANES data, which allowed for the inclusion of a broad sample of American adults from various age groups. However, after applying specific exclusion criteria for this analysis, the resulting study population may no longer fully reflect the national population. Nonetheless, this approach resulted in a substantial sample size and enabled us to account for several potential confounding factors, which greatly improves the reliability of our findings. Our statistical models extensively analyzed the correlation between individual CMI and heightened endometriosis levels. In addition, we created smooth curve fits and RCS to represent the link between the two variables, allowing us to examine the correlation from many angles and enhance the robustness of our findings.

Nevertheless, our study does possess certain constraints. Firstly, due to its cross-sectional nature, establishing a causal relationship between CMI and endometriosis is difficult. Furthermore, even after accounting for potential confounding variables, it is still possible that there are additional unmeasured confounding effects that have not been fully accounted for. Furthermore, many data in our study, such as smoking and drinking habits, are based on self-reporting, which could potentially create bias. Furthermore, our study relies on data collected from American adults, which raises doubts about the applicability of our findings to individuals from different countries and areas. Hence, it is imperative to conduct extensive prospective cohort studies to validate our findings.

The clinical significance of the observed association between CMI and endometriosis is noteworthy, particularly in terms of early identification, diagnosis, and intervention for patients with elevated CMI. This finding suggests that women with higher CMI, who are already at risk for metabolic and cardiovascular issues, may require closer monitoring for endometriosis. Furthermore, it raises the possibility of incorporating CMI as a marker in clinical guidelines for managing endometriosis patients. In particular, for those already diagnosed with endometriosis, heightened attention to cardiovascular health may be warranted, as they could be more susceptible to developing heart disease or other metabolic complications.Additionally, by integrating knowledge of the increased cardiovascular risk associated with higher CMI, clinicians could adopt a more comprehensive approach that includes lifestyle modifications, pharmacological interventions, and early preventive measures aimed at reducing the likelihood of cardiovascular disease. Such personalized treatment strategies could help mitigate long-term health risks, offering targeted care for women who are affected by both conditions.

## Conclusion

To summarize, our research shows an association between CMI and the likelihood of getting endometriosis. Given the high incidence of endometriosis and the unclear pathogenesis, further research is necessary to verify our findings and determine whether CMI could serve as a predictive indicator for endometriosis.

## Data Availability

The data were derived from sources in the public domain: NHANES https://wwwn.cdc.gov/nchs/nhanes/default.aspx.

## Glossary

CMI: Cardiometabolic Index
NHANES: National Health and Nutrition Examination Survey
NCHS: National Center for Health Statistics
CDC: Centers for Disease Control and Prevention
MEC: Mobile Examination Center
TG: Triglycerides
HDL-C: High-Density Lipoprotein Cholesterol
WHtR: Waist-to-Height Ratio
PIR: Poverty Income Ratio
BMI: Body Mass Index
OR: Odds Ratio
CI: Confidence Interval
SD: Standard Deviation
RCS: Restricted Cubic Splines
ER-β: Estrogen Receptor Beta
ER-α: Estrogen Receptor Alpha
NF-κB: Nuclear Factor Kappa-light-chain-enhancer of Activated B Cells
VEGF: Vascular Endothelial Growth Factor
FGF-2: Fibroblast Growth Factor 2
HGF: Hepatocyte Growth Factor
IGF: Insulin-like Growth Factor
TNF-α: Tumor Necrosis Factor Alpha
TGF-β: Transforming Growth Factor Beta
PlGF: Placental Growth Factor
TF: Tissue Factor
NPY: Neuropeptide Y
EGF: Epidermal Growth Factor
ASCs: Adipose Stromal Cells
