# Supplementary material for: Relationship between Cardiometabolic index and endometriosis in a US nationally representative sample: results from NHANES 1999-2006
Source: Front Endocrinol (Lausanne). 2024 Nov 12;15:1450965. doi: 10.3389/fendo.2024.1450965 (PMC11590797; doi:10.3389/fendo.2024.1450965)
Supplement: Supplementary file 1 [file Table1.docx]

Table S1: Comparison of Basic Characteristics Between Included and Excluded Participants in the Study from NHANES 1999–2006

| **characteristics** | **Included** | **Excluded** | **P for trand** |
| --- | --- | --- | --- |
| Sample size | 2274 | 18736 |  |
| **Age(years)** | 37.17±9.95 | 36.18±24.51 | 0.0111 |
| **BMI(kg/m^2^)** | 28.03±7.09 | 25.72±7.63 | ＜0.0001 |
| **waist circumference(cm)** | 91.99±16.00 | 86.00±20.00 | ＜0.0001 |
| **height(cm)** | 163.46±6.63 | 153.99±18.58 | ＜0.0001 |
| **Ethnicity,%** |  |  | 0.2703 |
| Mexican American | 7.69 | 8.48 |  |
| Other Hispanic | 5.81 | 5.89 |  |
| Non-Hispanic White | 68.83 | 67.30 |  |
| Non-Hispanic Black | 12.42 | 12.63 |  |
| Other Race | 5.25 | 5.69 |  |
| **Education level,%** |  |  | ＜0.0001 |
| Less than high school | 15.98 | 20.00 |  |
| High school | 23.24 | 24.94 |  |
| More than high school | 60.78 | 55.06 |  |
| **marital status ,%** |  |  | ＜0.0001 |
| Married/living with partner | 66.60 | 40.27 |  |
| Separated/divorced/widowed | 15.35 | 21.84 |  |
| Never married | 18.06 | 37.89 |  |
| **Smoking,%** |  |  | ＜0.0001 |
| Current-smoker | 52.84 | 46.30 |  |
| Non-smoker | 47.16 | 53.70 |  |
| **Drinking,%** |  |  | ＜0.0001 |
| Yes | 61.71 | 53.37 |  |
| No | 38.29 | 46.63 |  |

Notes: Data are presented as(%) or Mean ± SD .
